# A Life Course Study on Traumatic Brain Injury and Physical and Emotional Trauma in Foster Children

**DOI:** 10.1089/neur.2020.0054

**Published:** 2021-03-02

**Authors:** Michael D. Cusimano, Rachel Lamont, Stanley Zhang, Anamika Mishra, Melissa Carpino, David Wolfe

**Affiliations:** ^1^ Injury Prevention Research Office, Division of Neurosurgery, Li Ka Shing Knowledge Institute, Keenan Research Centre, St. Michael's Hospital, Toronto, Ontario, Canada.; ^2^ Faculty of Medicine, University of Toronto, Toronto, Ontario, Canada.; ^3^ Dalla Lana School of Public Health, University of Toronto, Toronto, Ontario, Canada.; ^4^ Centre for School Mental Health, Faculty of Education, Western University, London, Ontario, Canada.

**Keywords:** childhood abuse, foster children, life course, normalization, traumatic brain injury, trauma

## Abstract

Foster children are exposed to high levels of abuse, violence, and other adverse events throughout their childhood and adolescent years. Forms of brain injury, notably traumatic brain injury (TBI), are understudied in the foster child population. This study aimed to explore different forms of brain injury and their cognitive, behavioral, and psychological/emotional effects on current and former foster children using a life course perspective. A thematic analysis with a life course perspective was used to examine semi-structured, open-ended interviews conducted with current and previous foster children between the ages of 16 and 29 years. The study included 47 participants: 25 males (53%) and 22 females (47%) with an average age of 21 years and an average of 11.2 years of education. Of 47 current and previous foster children between the ages of 16 and 29, two-thirds had sustained one or more TBIs. Through a thematic analysis, four overarching and inter-related themes emerged from the data: frequent TBI, normalization (of abuse, violence, injury, and neglect), emotional trauma, and dangerous coping methods such as alcohol use in 94% and recreational drug use in 81%. Normalization of adverse events, emotional trauma, and the use of dangerous coping methods occurred in 66%, 81%, and 49% of participants, respectively, and are the cumulative toxic long-term effects of early negative life experiences and repeated forms of brain injury. Early and continued exposure to TBI, abuse, violence, and/or neglect with continued maladaptive behaviors suggests that the participants may have experienced changes in brain structure and function over their lives that provided the milieu for continued vulnerability to personal and future injury to future generations. These behavioral and perceptual changes point to a toxic combination of injuries that result in continued vulnerability to repeated injury through contextual exposure to risks and maladaptive normalization, emotional trauma, and risky coping styles.

## Introduction

Foster children are vulnerable members of society, often exposed to high levels of violence and abuse^1–7^ throughout their developmental years. Trauma exposure among youth in foster care ranges from 80 to 97%, with many reporting adverse family experiences including neighborhood violence, caregiver violence, and living with a mentally ill caregiver or someone with an alcohol or drug problem.^8^ The often-turbulent family lives and increased exposure to adverse childhood experiences (ACE) render foster children at risk for developing physical, behavioral, emotional, and developmental health problems,^2,9,10^ as well as various mental health issues.^6–10^ It is therefore not unreasonable to hypothesize that traumatic brain injury (TBI) would be a frequent and important form of brain injury in this group; but research on this is lacking.^11–15^ Although many behavioral and psychological problems have been studied in foster children, literature on TBI in this population is limited.

TBI may be defined as a bump, blow, or jolt to the head that disrupts normal brain functioning, leading to an altered mental state, such as a loss of consciousness or being dazed and confused.^16^ Child abuse and neglect have been reported as predisposing factors to TBI.^17^ Given that foster children report higher levels of physical and sexual abuse, violence, and maltreatment than the general population,^1–7^ foster children may be at an increased risk for sustaining injuries, including TBI. Studies also report that foster children are at risk for developing substance use and alcohol use disorders and experiencing increased aggression,^7^ with childhood trauma found to be related to substance use disorders at an earlier age^18^ and experiences of physical abuse found to be associated with higher aggression levels.^19^ These negative outcomes have also been found to be associated with TBI.^7^ Given the unique life experiences and vulnerable nature of this population, the impact of TBI on foster children merits further investigation.

Results from a growing number of reports in the literature reveal that TBI in childhood or adolescence may be associated with violent and antisocial behavior, vocational failure, long-term cognitive deficits, impaired executive functioning, substance misuse, neurodegenerative diseases, socioeconomic decline, and homelessness in adulthood.^20–29^ One in five adolescents have sustained a TBI and one-third of adolescents who acquire a TBI have additional TBIs later in life.^21,30^ Studies have further reported high rates of injury among inner city youth^31^ and high rates of TBI among youth entering the correctional system,^32,33^ with offenses tending to occur following TBI, rather than pre-injury.^34,35^

Brain injury and dysfunction can also occur through exposure to abuse and other adverse events. Growing evidence supports a relationship between childhood exposure to abuse, trauma, and numerous neurobiological deficits.^36–40^ For instance, increased stress has been observed to inhibit the ability of the hippocampus to grow new neurons during adulthood.^41,42^ In adults, smaller hippocampal volume has been associated with the occurrence of early-abuse post-traumatic stress disorder (PTSD).^43–45^ PTSD in children has been linked to smaller whole brain and corpus callosum volume.^46,47^ Childhood abuse and depression in women is also correlated with increased cortisol reactivity to stress.^48,49^ Thus, traumatic events often experienced by foster children may be considered forms of brain injury, as exposure to such events in childhood and adolescence has the capability to alter the brain structure and function throughout the life course.

The life course perspective is a theory for understanding how social influences work together to create biographical patterns and shape human development.^50^ In life course research, attention is paid to temporal patterns in both individual and social change, as well as the influence that an individual's environment has on their development.^50^ The life course perspective posits that family, community context, and social institutions influence human biographies throughout life, and acknowledges how lives change over time in relation to these external influences.^50^ Previous life course research has found that child removal and foster care can be traumatic for children, as they often encounter unexpected, non-normative transitional periods.^50^ Our study will speak to how the temporal sequence of ACE may influence the brains of foster children throughout their lives.

This qualitative study using a life course perspective was undertaken to explore TBI and the relations of forms of brain injury caused by physical force and/or traumatic events, and the cognitive, behavioral, and psychological and emotional effects on current and former foster children in Canada.

## Methods

### Study design, ethics approval, and participants

Participants who had been foster children were recruited from Yonge Street Mission Evergreen Centre for Street Youth, Youth Unlimited, the Catholic Children's Aid Society, and Covenant House in Toronto, Ontario, Canada. Potential participants were screened for foster status and ability to speak English. Participants were excluded if they suffered a TBI within the 3 months prior to recruitment, if they were visibly intoxicated, if they were not or had not been designated as a foster child by the province of Ontario, or if they were unable to provide informed consent. In total, 139 expressed interest in the study, out of which 83 participants were enrolled and 36 withdrew or were excluded due to incomplete interviews. Ethics approval was granted by the Research Ethics Board of St. Michael's Hospital, Toronto, Ontario, Canada.

### Data collection

Semi-structured, open-ended interviews were conducted with 47 participants based on a predetermined interview guide; however, topics were explored as they arose. The average interview duration was 1 h, allowing participants to further elaborate on topics. Interviews were audio recorded with the verbal and written consent of the participants. Participants were defined as TBI-positive if in their lifetime they had experienced a hit to the head resulting in any of the following: (1) loss of consciousness, (2) loss of memory, or (3) alteration in mental state at the time of the TBI (e.g., feeling dazed or confused).^16,51^

We utilized the Brain Injury Screening Questionnaire (BISQ) and the Adverse Childhood Experiences (ACE) questionnaire to further explore participant experiences and confirm interview findings. The BISQ was developed as a TBI screening tool to document lifetime history of self-reported TBI and the presence of symptoms.^16^ The BISQ also seeks to rule out alternative explanations for symptoms, and reports on the probability that problems in daily functioning are due to brain injury.^16^ The ACE questionnaire measures experiences such as abuse, neglect, and household dysfunction, and posits that these detrimental childhood experiences are cumulative, and can be developmentally disruptive in numerous ways.^52^

### Statistical analysis

Interviews were transcribed and imported into the NVivo software (QSR International, Australia). The data were analyzed using modified steps of thematic analysis, as outlined by Braun and Clarke.^53^ The first step in the thematic analysis was for analysts to familiarize themselves with the data through reading the transcripts and searching for broad meanings and patterns. Analysts also had access to a pre-existing codebook that had been developed through analysis of other interviews of vulnerable persons with TBI in several associated research studies. The second step was to utilize the existing codes, as well as generate additional codes, to organize qualitative data into meaningful groups based on the mention of critical information. During this process, two coders (R.L. and A.M.) independently coded 15% of all transcripts and calculated a Cohen's Kappa value of 0.42. Discrepancies between coders were discussed extensively and resolved, and the next 15% of the transcripts were coded. An acceptable Cohen's Kappa value of 0.80 was attained.

The remaining 33 transcripts were split and independently coded, comparing and re-calculating a Cohen's Kappa value every fifth transcript for coding consistency. The third step involved identification of themes. To do so, connections and relationships between codes were assessed and organized into prominent themes. The fourth step involved reviewing themes to ensure data were accurately classified and clear distinctions existed between each theme and the respective data. Upon completion, the fifth step was to clearly define what each theme encompassed and label themes accordingly. Lastly, the two coders analyzed and explored themes and the interconnections between each to create a cohesive story of the rich qualitative data present. Summaries for each participant outlining significant life events in chronological order were also written alongside the coding process for a deeper understanding of each participant's personal story. To examine the temporal patterns of events and the influence of associated events, family context, community context, and social institutions throughout life, a life course perspective was used as a framework to explore how lives changed over time in relation to these external influences.^50^

## Results

### Participant demographics

As shown in Table 1, the final study sample included 47 participants: 25 males (53%) and 22 females (47%) with an average age of 21 years and an average of 11.2 years of education. The study sample was predominantly Caucasian (44.7%) and African-North American (25.5%) (Table 1). Overall, 66% of participants (*n* = 31) reported sustaining at least one TBI in their lifetime (Table 1).

**Table 1.** Demographic Characteristics of Study Participants (*n* = 47)

Variables	Total (*n* = 47)
Sex	
Male	25 (53.2%)
Female	22 (46.8%)
Age (years)	
16–19	14 (29.8%)
20–23	21 (44.7%)
24–27	9 (19.1%)
28–29	3 (6.4%)
Race	
Caucasian	21 (44.7%)
African-North American	12 (25.5%)
Hispanic	2 (4.3%)
First Nations/Native-American	2 (4.3%)
Asian-American	2 (4.3%)
Other	8 (17.0%)
Education (years)	
0–4	0 (0%)
5–8	3 (6.4%)
9–12	35 (74.5%)
13–17	5 (10.6%)
Unspecified	4 (8.5%)
TBI status (interview report)	
1+ TBI	31 (66.0%)
No TBI	16 (34.0%)

TBI, traumatic brain injury.

### Thematic analysis

**FIG. 1.**
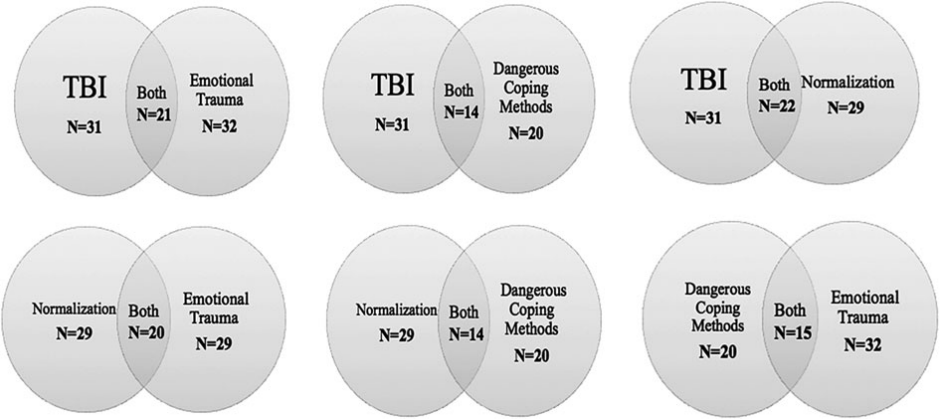
Relationship between themes. N = sample size. TBI, traumatic brain injury.

Four overarching themes emerged through analysis of the interview data: (1) TBI, (2) normalization, (3) emotional trauma, and (4) dangerous coping mechanisms (Table 2). The inter-relatedness of these themes is outlined in Figure 1. Further details about the themes are provided as follows.

**Table 2.** Thematic Analysis

Themes	Sub-themes
Traumatic brain injury	Time of TBI
	Mechanism of TBI
	Repeated TBI
	Impact of TBI
Normalization	Normalization of abuse
	Normalization of violence
	Normalization of injury
	Normalization of neglect
Emotional trauma	Avoidance and inability to cope
	Counseling and psychiatry
	Mental health issues
Dangerous coping methods	Alcohol abuse
	Recreational drug abuse
	Self-harm

TBI, traumatic brain injury.

### TBI was common among participants

As reported in Table 1, 66% of participants (*n* = 31) had at least one TBI in which they experienced being dazed, confused, dizzy, having memory loss, or loss of consciousness. Multiple participants reported more than one TBI throughout their lives, equaling a total of 55 TBIs reported among 31 participants.

#### Time of TBI

Of the 31 participants who reported at least one TBI, 41.9% (*n* = 13) had one or more TBIs during their childhood years (0–12). A 22-year-old female participant who was put into numerous foster homes and severely sexually and physically abused reported two TBIs in her early years. After her second TBI, she experienced intense headaches, as well as dizziness and double vision, which she still suffered from at the time of the interview. She stated:

*“I have been sexually abused before, and they hit me with a bat in the head when I was 6 and 7, and I would get a headache every time.”*

As well, 51.6% (*n* = 16/31) of these participants reported at least one TBI throughout their teenage years (13–19). A 26-year-old male who suffered physical and emotional abuse for 14 years discussed having a crystal meth addiction and demonstrated normalization of violence. The participant suffered three TBIs in his lifetime and reported he felt he was still recovering from the third TBI, which was the result of a violent altercation. The participant stated:

*“When I was 19 and I got punched in the nose … so I ended up falling backwards and like almost breaking my back and hitting my head on the rock.”*

In total, 32.3% (*n* = 10/31) of participants reported at least one TBI in their 20s (20–29). A 21-year-old female with a severely abusive mother and a history of sexual abuse reported two TBIs, both from physical abuse. The participant reported feeling dazed, confused, and experiencing headaches after both TBIs, and noticed mood changes and tiredness after the second. She explained:

*“Happened actually when I got my first place with uh, now my ex-fiancé. The fights were always bad between him and I, but the last fight we had before we ended was the worst. We got into a pretty physical fight. He ended up pushing me and I ended up hitting my head off of a rock as well as the ground.”*

#### Mechanisms of TBI

Of the 55 TBIs, two mechanisms accounted for half of all TBIs: physical abuse, usually as a child beaten by a caregiver or from a domestic partner (*n* = 14; 26%), and violent altercations with strangers or friends, but not intimate partners or caregivers (*n* = 14; 26%). Caregiver and intimate partner violence were also common themes. A 24-year-old female participant with manic-depressive and bipolar disorder, as well as a history of physical abuse and incarceration, reported two TBIs in her lifetime. In the first incident, she was hit in the head with a metal baseball bat at age 8 and lost consciousness. An incident of physical abuse by her ex-boyfriend led to the second TBI, causing her to forget who she was for 3 days. She shared:

*“He had me off my feet. He head-butted me and the back of my head hit the wall and his head-butt made a goose egg right here. And then he grabbed me by the side of my face like this—like the whole entire side of my face like that, my whole entire skin with cheeks and everything and just punched me in the side of the temple twice.”*

Likewise, 26% (*n* = 14/55) of all TBIs across participants were caused by a violent altercation. Violent altercations included gang fights, fights with friends, and fights with strangers in locations such as bars or on the streets. A 24-year-old male participant with a history of gang involvement, dealing drugs, and an abusive and neglectful childhood, reported two TBIs. After his second TBI, the participant required medical attention and suffered from headaches and double vision, which had still not subsided years later. He stated:

*“I have been hit in the head and have been dazed and confused but I have been knocked unconscious when I was in jail. I was in a fight. And so I got a couple of blows to the head and so one hit the temple and left me unconscious.”*

Other mechanisms of TBI among participants included “accidental” falls (20%, *n* = 11/55), sports-related injuries (9.1%, *n* = 5/55), other “accidents” (9.1%, *n* = 5/55), workplace injuries (5.5%, *n* = 3/55), and motor vehicle accidents (5.5%, *n* = 3/55). A 16-year-old female reported two TBIs in her lifetime; after the first TBI, she could not speak properly and had continuous headaches. She stated:

*“I was locked out of my house, so I thought it would be a good idea to go through my bedroom window and we lived in the basement at that time so it was an 8-foot drop onto ceramic tiles and I lost my footing and smashed my head off the ground.”*

A 24-year-old male with a history of violence and incarceration, and a mother who drank alcohol during pregnancy, reported two sports-related TBIs. The first incident occurred during a hockey game at age 8, in which he was body-checked and hospitalized with a concussion. The second occurred during a mountain-biking competition, resulting in the participant spending weeks in the hospital. He said:

*“I was in a competition and I was going down a mountain and … I took the tree and I flew off the tree and I wrapped myself around the tree. I was probably going 30 kilometers an hour.”*

#### Repeated TBI

Of the 31 participants reporting a TBI, 48.4% (*n* = 15) experienced subsequent TBIs later in life. Nine participants reported two TBIs, three participants reported three TBIs, and three participants reported four TBIs. A 26-year-old male with a violent and abusive past reported three TBIs in his lifetime, two of which were the result of violent altercations. The participant was previously hospitalized with a mild concussion and reported feeling lightheaded after hits to the head. Discussing his repeated TBIs, the participant stated:

*“Probably about three to four … just like a couple from getting punched in the head.”*

#### Impact of TBI

Some participants were able to reflect on the impact of their TBI on their experiences and choices later in life. Experiencing a TBI could result in both voluntary and involuntary changes in behaviors, feelings, or perceptions. Voluntary changes seen across participants included going out in public less and being careful about the people they trust. Involuntary changes included a decline in overall happiness, problems sleeping, as well as mood swings and aggressive tendencies.

A 25-year-old male with a physically abusive father discussed growing up in a community riddled with poverty, gang violence, drugs, and weapons. The participant reported two TBIs due to violent altercations, resulting in throbbing in his head, feeling dazed, and experiencing headaches. Reflecting on the changes caused by his TBI, the participant stated:

*“Well, I would say the last head injury that I had I just stopped going out because I have these migraines now and they are not fully explained. They just come out of nowhere sometimes and they last for like weeks and stuff so uh, I don't really go out much anymore because of that.”*

A 22-year-old female with at least three TBIs reported being hospitalized due to sharp pains in the back of her head, as well as blurred vision, memory problems, and multiple instances of blacking out related to her head injuries. The participant reflected on how her TBIs affected her patience and aggression, stating:

*“I was always the most calm, quiet person where I don't go into people's business, I just do my own thing. But lately I've been, for 3 years, I have been like that I get mad easily and just get annoyed really, really easily. I just get set off quickly.”*

### Participants had a tendency to normalize adverse events

Normalization refers to attitudes and cognitions indicative of desensitization to adverse events. It is a subconscious process, often used to prevent emotional distress, while consequently legitimating troubling behavior.^54^ Of all 47 participants, 66% (*n* = 31) demonstrated signs of normalizing adverse events.

#### Normalization of injury

Of these 31 participants, 51.6% (*n* = 16) demonstrated normalization of injury. Normalization of injury was the most common form of normalization, as many participants often failed to understand the impact or severity of injuries. An 18-year-old female participant with severe anger issues reported growing up in a family that would abuse her physically and not take her to the doctor when she was injured. Demonstrating normalization of injury related to how she grew up, the participant stated:

*“My family's very like ‘if you're not bleeding to death then you don't go to the hospital’… It's just like, ‘suck it up and tough it out.’”*

Normalization of injury was also demonstrated by an 18-year-old male with an abusive father and a mother who was addicted to drugs. Speaking of an incident where his friend was in serious danger, the participant said:

*“This guy didn't listen to me and I told him before you take a step, pat the ice and he didn't pat the ice and so he went and slipped and bust himself, fell under the water and everything and could've drowned. Hah. Crazy … I just laughed at him [laughs].”*

#### Normalization of abuse

Of the 31 participants who showed signs of normalization of adverse events, 35.5% (*n* = 11) appeared to normalize abuse. Normalization of abuse was seen in a 20-year-old male participant with a history of extensive physical abuse and neglect by his aggressive, alcoholic mother. The participant stated:

*“One of my rave sisters stays at this guy's house and the usual happened, you know? Tried to rape her and shit, you know? … Tried to, no big deal though.”*

A 21-year-old male with a mother who was addicted to drugs and worked as a prostitute reported being physically abused by his mother's boyfriend and suffering multiple TBIs in his lifetime. The participant demonstrated the belief that abuse was a way of making him stronger. He shared:

*“She called me a big baby and basically took a belt to me and locked me in a room for a day with no food … My mum always beat me if I cried, if I did anything emotional, my mum tried to teach me to be solid as a rock.”*

#### Normalization of violence

Of the 31 participants showing signs of normalization, 45.2% (*n* = 14) appeared to normalize violence or violent acts. A 23-year-old male with an alcoholic mother and a sexually abusive father reported being involved with gangs and suffering 10 or more head injuries as a result of violent altercations. Demonstrating desensitization to violent acts, the participant stated:

*“All I know I got—when they were in mid-kick I took them by the other ankle and went like that [pulled their stable ankles so that they would lose balance], they both fell and smacked their head on the rocks. I got up and started laughing at them and walked away.”*

A 24-year-old male with an abusive, neglectful, and gang-involved father demonstrated aggressive tendencies and appeared to normalize violent events he had been involved in. He said:

*“I don't really consider stabbing really serious because it didn't go through and through.”*

#### Normalization of neglect

Normalization of neglect was the least common form of normalization; 12.9% (*n* = 4/31) of participants normalized neglect received throughout their childhood and teenage years. A 21-year-old male with a history of TBI reported physical, sexual, and emotional abuse, and shared that his father and stepmother neglected him growing up. The participant stated:

*“I didn't get anything for dinner. I just had to starve. But oh well.”*

A 20-year-old male participant with an alcoholic and physically abusive mother reported that his mother was often too intoxicated to bring him to the doctor after an injury or provide adequate daily care. The participant showed signs of normalizing neglect, stating:

*“I've literally like … never had a parent … how can I … there is no such thing as neglect … it's just life.”*

### Many participants demonstrated signs of emotional trauma

Traumatic experiences and associated emotional states can trigger or reinforce negative beliefs of the individual about themselves and their environment. This may cause increased susceptibility of stress responses by changing the regulation of the hypothalamic-pituitary-adrenal axis, thus affecting brain function and behaviors, and increasing the risk of developing psychopathological disorders.^55^ In total, 80.9% of participants (*n* = 38/47) demonstrated signs of emotional trauma.

#### Avoidance and inability to cope

Of the 38 participants who demonstrated signs of emotional trauma, 84.2% (*n* = 32) avoided questions asked by the interviewer, declined to answer altogether, or expressed that they were still coping with events that occurred in their past. A 24-year-old male participant with an absent father and a mother who was addicted to cocaine demonstrated signs of emotional trauma. The participant avoided answering questions regarding his past, and expressed that he believes he has abandonment issues. He shared:

*“I can't really say too much on it, I'd rather pass on that question. There's a lot of demons in my closet that I had to come to terms with, by myself, without anybody around.”*

Similarly, emotional trauma was demonstrated by a 22-year-old female with multiple TBIs and a history of physical abuse by her parents, as well as sexual abuse by her cousin and his friends. The participant shared:

*“I've seen a lot of things. Some of them are like scars, like I think about it every day, but, um … I try to forget it.”*

#### Counseling and psychiatry

Nearly 45% of participants showing signs of emotional trauma (*n* = 17/38) previously or currently attended counseling or psychiatry sessions. A 21-year-old female with two TBIs and violent tendencies reported being in multiple physically abusive relationships, being physically abused at a young age, and visiting a psychiatrist. The participant stated:

*“I didn't start seeing the psychiatrist until about 6 months after my domestic. I started seeing my psychiatrist because I started noticing—as I said—different things were going on with me that I had not experienced before.”*

A 24-year-old male who was neglected and physically abused growing up struggled to speak about his experiences with anyone aside from his counselor. The participant shared:

*“I guess because I'm still talking about it with some people and stuff like that … my counselor and stuff like that. It's not one topic I like to bring up because neglect is like abuse too right, so it's another topic I didn't want to bring up because now that I think about it, it makes me upset right … that I grew up that way.”*

#### Mental health issues

Among the 38 participants with signs of emotional trauma, 86.8% (*n* = 33/38) reported mental health issues. These issues included diagnosed mental illnesses, as well as some cases of self-reported mental health conditions. Signs of having aggressive tendencies was not considered to be a mental health issue for the purpose of this analysis. Moreover, 21.2% (*n* = 7/33) of participants with a mental health issue reported having anxiety. A 24-year-old male with a history of TBI, alcohol abuse, and criminal activity shared:

*“They had warrants out for my arrests and during that time I was drinking extremely heavy and developed anxiety through it.”*

Among the 33 participants who reported mental health issues, 45.5% (*n* = 15) reported depression or depressive symptoms. A 21-year-old female participant who reported three TBIs, as well as a history of alcohol abuse and self-harm, discussed changes in her mental state after being sexually abused by her uncle. The participant stated:

*“I used to be happy, jolly, you know? Gets along with everybody, talked to everybody, but since this started happening … I have gone from happy to not so happy, to depressed, to being anti-social, then kind of getting defensive and then I kind of broke down.”*

The remaining 33.3% (*n* = 11) of these participants reported various other mental health issues, including PTSD, split personality disorder, and schizophrenia. A 24-year-old female with two TBIs, as well as a history of alcohol abuse and drug addiction reported suffering from a mental illness. She said:

*“The SWAT team would have to try their hardest to get me off of somebody I wanted to kill okay? … I'm schizophrenic. I could lose it and really hurt you.”*

### Participants used maladaptive, dangerous, or risky coping mechanisms

Overall, 48.9% of all 47 participants (*n* = 23) reported using maladaptive, dangerous, or risky coping methods. Attempting to cope using alcohol, other substances such as recreational drugs, or self-harm were considered to be dangerous for a participant's well-being.

#### Alcohol use

Among all 47 participants, 93.6% (*n* = 44) currently or previously used alcohol. Of these participants, 40.9% (*n* = 18) reported using alcohol as a method to cope with various stressors. A 21-year-old female with a history of TBI and physical abuse reported beginning to drink at the age of 13 and using alcohol as a method to cope with emotional trauma. The participant shared:

*“It became a way for me to get rid of the pain and everything that I was going through, whether it be the physical pain or the mental and emotional pain that I was going through and it just became a way of coping for me. Unfortunately, maybe it was the wrong way to cope, but it was the only way I could cope.”*

Similarly, a 16-year-old female who had been physically abused and showed signs of emotional trauma, as well as normalization of abuse and violence, also reported using alcohol to make herself happier and avoid feeling stressed. She shared:

*“I think there were certain moments in my life where I notice I got into problems, or I got too stressed out, I drank. I noticed that after I was done with my boyfriend because it was like when I wasn't drinking, I felt like I wasn't doing anything. I felt like I wasn't happy.”*

#### Recreational drug use

Recreational drug use was common across participants, with 80.9% (*n* = 38/47) reporting using drugs currently or previously. Of the participants who used drugs, 47.4% (*n* = 18/38) turned to drugs as a way to cope with adverse events. The use of recreational drugs as a coping mechanism was demonstrated by a 26-year-old female with a history of physical abuse and violence. She stated:

*“I just basically tried to use drugs and alcohol to cope with my situations at home and learn to distance myself emotionally from my family.”*

The tendency to cope by using recreational drugs was further demonstrated by a 17-year-old female with an alcoholic father who dealt drugs. Her father physically abused her, giving her a TBI at a young age. She said:

*“I started using needles again this year and that's when I started realizing why I did it, because it was a way of self—just like release of stress and everything—and just like the drug that I was doing with it was just like an excuse to be able to do it.”*

#### Self-harm

Of all 47 participants, 17% (*n* = 8) reported attempting to harm themselves at one or more points in their lives. A 21-year-old female with a history of TBI and physical abuse reported attempting suicide as a result of sexual abuse by her uncle. She shared:

*“I was harming myself with a razor blade. I was trying to overdose on my medication. I was on Ritalin … I was making up excuses to think of every single possible thing just to get out of that house. Even running away or starting fires, that was the way … of how scared I was. I needed to do something.”*

**FIG. 2.**
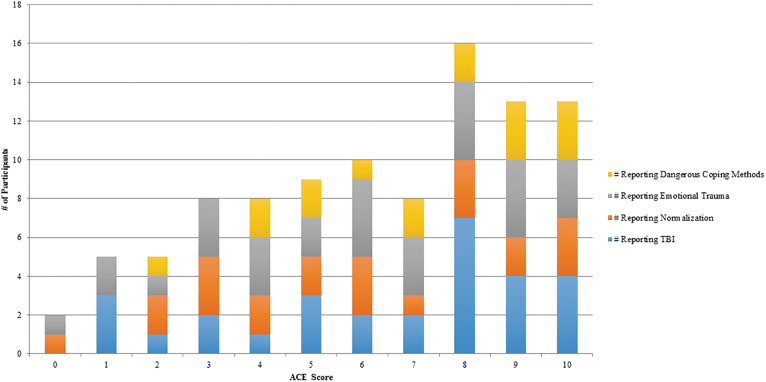
ACE results. ACE, adverse childhood experiences; TBI, traumatic brain injury.

Table 3 and Figure 2 show the ACE results from our population. Table 4 contains the BISQ results of participants.

**Table 3.** Adverse Childhood Experiences (ACE) Questionnaire Scores

Number of adverse childhood experiences	Number of total participants	Number reporting TBI	Number reporting normalization	Number reporting emotional trauma	Number reporting dangerous coping methods
0	2	0 (0%)	1 (50.0%)	1 (50.0%)	0 (0%)
1–2	5	4 (80.0%)	2 (40.0%)	3 (60.0%)	1 (20.0%)
3–4	9	3 (33.3%)	5 (55.6%)	6 (66.7%)	2 (22.2%)
5–6	7	5 (71.4%)	5 (71.4%)	6 (85.7%)	3 (42.9%)
7–8	10	9 (90.0%)	4 (40.0%)	7 (70.0%)	4 (40.0%)
9–10	9	8 (88.9%)	5 (55.6%)	7 (77.8%)	6 (66.7%)

TBI, traumatic brain injury.

**Table 4.** Brain Injury Screening Questionnaire (BISQ) Scores (*n* = 45)

BISQ result	N
Negative probability	14
Low probability	18
Moderate probability	9
High probability	4

## Discussion

We identified high rates of TBI (66%) and a series of adverse experiences of abuse, neglect, and violence beginning in childhood in the lives of foster children. The themes of frequent TBI, normalization, emotional trauma, and maladaptive coping styles were intricately connected to one another. When viewed holistically over the life course, these interconnected themes reveal a temporal sequence of repeated TBI and other forms of brain injuries that created a vulnerability and continued high risk for repeated injury, addiction, and risky behaviors associated with adverse health and social outcomes later in life (Fig. 1).

The striking finding of this study was that these themes are so highly interconnected and that they never occur in isolation (Fig. 1). For example, of the participants reporting at least one TBI in their lifetime (*n* = 31), 71% (*n* = 22) also showed signs of normalization of adverse experiences (injury, abuse, violence, neglect), 67.7% (*n* = 21) also experienced emotional trauma, and 45.2% (*n* = 14) also reported the use of dangerous coping mechanisms (alcohol, recreational drugs, and self-harm). Interestingly, of all participants who reported a TBI, 32.2% (*n* = 10) demonstrated normalization, emotional trauma, and dangerous coping methods. Although these findings cannot determine causality, their temporal nature and interconnections clearly support a body of literature that shows adverse events in childhood and/or adolescence can be associated with adverse outcomes later in life.^20–29^ Our results highlight that TBI is a key event in life trajectories and the connections seen between themes lead to a state of vulnerability in foster children.

Previous research shows that each ACE increases the likelihood for early initiation of drug use 2- to 4-fold, and individuals with five or more ACE were 7- to 10-fold more likely to report drug use or addiction later in life.^52^ Only two participants did not report any ACE.

Our results also support prior literature that shows that children and adolescents who are homeless and/or entering the correctional system have high rates of TBI^32,33,56^ and that the high rates of abuse and violence that foster children are exposed to may also put them at an elevated risk of TBI.^1–3^ Our BISQ data indicate that 29% have a moderate or high probability that problems in daily functioning were due to a brain injury (Table 4), and further highlight that TBI can interact with other forms of brain injury that contribute to adverse behaviors and outcomes in foster children. Our data shows that these individuals live and grow up in abusive and/or violent environments, creating susceptibilities to further intentional injuries interconnected with the establishment of maladaptive beliefs and behaviors, such as normalization of violence, and drug and alcohol abuse.

These maladaptive behaviors establish a context for continued cycles of vulnerability to further injury and adverse outcomes. As a means to break this cycle, others have shown that positive home environments provide protection following a TBI, and family environment has the ability to moderate the psychosocial outcomes of TBI in youth.^57^ Thus, one approach to break this negative cycle of interconnected behaviors and beliefs might be to provide the means for creating positive family environments early in life, before repeated injuries occur and lead to the establishment of maladaptive behaviors. Whether this would take the form of support to biological parents or foster parents is beyond the scope of this article.

Our findings support previous literature establishing that brain injuries of this nature can lead to cognitive and behavioral changes later in the life course.^20–29^ Our study adds to this literature by not only exploring the life circumstances leading up to TBI, as well as the various changes that can occur after TBI, but also other forms of brain injury that may go unseen. The most salient and novel findings of our study considered various adverse events in parallel with potential cognitive and behavioral changes that may be caused by TBI and other brain injuries, such as emotional trauma, normalization, and the use of dangerous coping methods.

Many of our findings also align with previous research on foster children and inner-city youth. Existing literature reports that inner-city youth can often view physical assault as a normal part of their childhood or adolescent years.^58^ Our research affirms these findings, as nearly half of the participants showed signs of normalizing violence (45.2%) and over one-third normalized abuse as well (35.5%). Normalization of violence may allow youth to disengage or distance themselves from their surroundings. Research also suggests how such normalization of violence may be a form of pathological adaptation resulting in desensitization to violence, a way for youth to cope with violence.^54^ Although normalization may serve to desensitize the witness or victim and cause an immediate decrease in emotional distress, it may also cause an increase in violent behavior in later years.^54^

Additionally, 84.2% of participants displayed signs of being unable to cope with past events in their lives, and 48.9% used dangerous or risky coping methods. These findings align with previous literature reporting on the use of negative coping methods in the foster child population. Garrido and colleagues found that the relationship between community violence exposure and experience of trauma symptoms, such as PTSD, depression, and anxiety was partially mediated by negative coping strategies such as self-harm, avoidance, and interpersonal aggression.^59^ Our work shows that these are intricately interwoven in the behaviors of these individuals and reinforced by life events and contexts. Ellerman further notes that foster children may develop self-destructive or ineffective coping strategies to deal with stresses.^12^ These points have multiple implications in youth who access the health care system or other systems, such as the criminal justice system. Rehabilitation of these individuals ought to take into consideration these prior forms of brain injuries along with the sets of maladaptive beliefs and behaviors to avert future adverse health or social outcomes. In addition, our results that show the interconnected web of adverse experiences and TBI, social and emotional injury, with maladaptive coping, drug use, self-harm, and mental health effects make it plausible to hypothesize that these events and effects could also manifest in the children of these individuals unless systematic strategies to prevent them are undertaken.

### The life course perspective

Epidemiological evidence is increasingly supporting the link between early life events and health at later life stages, often taking a life course perspective when considering health outcomes.^60^ Nearly half of all participants who reported a TBI experienced subsequent TBIs later in their lives. Forty-two percent of TBI-positive participants reported one or more TBIs in their childhood years (0–12). These findings may point to a link between early TBI and subsequent brain injuries. The interconnected maladaptive behaviors we have demonstrated provide a mechanism by which these individuals may be at a higher risk for future brain injuries and supports prior literature on recidivism in TBI.^21,30^

The life course perspective we have taken shows the many ways in which early-life experiences can shape health across a lifetime and across generations. Many of our participants commented on experiences of TBI early in life along with other forms of physical, psychological, and sexual abuse and neglect from adults in their lives. These adults were often biological caregivers, their partners, or foster caregivers. This frequently led to repeated cycles of continued violence, injury, and adverse behaviors and beliefs. In fact, of the 13 participants in our cohort who reported being in a physically abusive romantic relationship, 77% (*n* = 10) were also physically abused by their parent or caregiver in their childhood and/or adolescent years. Our data show evidence that these events can lead to a continuous cycle repeated over generations of adverse contexts and health and social outcomes. Breaking such generational cycles requires attention to the roles of repeated TBI and other forms of brain injury.

Nearly 90% of our participants with signs of emotional trauma reported mental health issues, and about half also required some form of counseling or psychiatric help in their teenage and/or adult years. The participants in our study also vividly recounted how interconnected and temporally related these forms of repeated childhood injury were with health outcomes, and potential brain changes manifested in mental health issues during the teenage and adult years. This supports prior life course research that also found that behavioral and mental health issues in adulthood have been linked to early adverse and traumatic experiences.^60^ Foster children typically leave home and enter the foster care system in a critical transitional period, and the traumatic events often experienced by foster children throughout their early years may contribute to the formation of emotional trauma symptoms and various mental health issues as they age. The prominence of the dangerous coping theme we identified that negatively affected participants over the course of their lives could be a potential avenue for intervention. Without robust social and cognitive coping systems, it is not difficult to see that these individuals could have future adverse health and social outcomes and contribute to similar events and outcomes in their own children. Developing strategies to address these points and the prevention of TBI, which is a known risk factor of adverse mental health outcomes, is another implication of our work.

### Limitations

This study has limitations. Our results are based on self-reported data, which may be prone to recall and other forms of bias. However, through detailed interviews, participants pieced together rational accounts of their lives and the vivid interconnectedness of events, particularly early life experiences. The questionnaire data also supported their recounting of events. Although the nature of injuries may have impaired the memories of some, the detailed participant summaries allowed us to determine the temporal sequence of events. Finally, although the nature of our study precludes causal inferences, it has provided a number of hypotheses that can be tested in future longitudinal work.

## Conclusion

The early and continued exposure to TBI, abuse, violence, and/or neglect with continued maladaptive behaviors suggests that the participants may have experienced changes in brain structure and function over their lives that provided the milieu for continued vulnerability to personal injury and for possible injury to future generations. These behavioral and perceptual changes point to a toxic combination of injuries that result in continued vulnerability to repeated injury through contextual exposure to risks and maladaptive normalization, emotional trauma, and risky coping styles. The results suggest that measures to prevent cycles of adverse events that start early in life may prevent a wide range of adverse health, emotional, and social events in individuals and also potentially prevent the appearance of such adverse outcomes in future generations.

## Abbreviations Used

ACE: adverse childhood experience
(ACE) questionnaire: Adverse Childhood Experiences questionnaire
BISQ: Brain Injury Screening Questionnaire
PTSD: post-traumatic stress disorder
TBI: traumatic brain injury
